# Burden of Malnutrition among Children and Adolescents with Cerebral Palsy in Arabic-Speaking Countries: A Systematic Review and Meta-Analysis

**DOI:** 10.3390/nu13093199

**Published:** 2021-09-15

**Authors:** Sami Mukhdari Mushta, Israt Jahan, Risad Sultana, Sarah McIntyre, Al-Mamoon Badahdah, Nihad A. Almasri, Catherine King, Harunor Rashid, Nadia Badawi, Gulam Khandaker

**Affiliations:** 1 Public Health Authority, Riyadh 13354, Saudi Arabia; smus9514@uni.sydney.edu.au; 2 The Children’s Hospital at Westmead Clinical School, The University of Sydney, Westmead, NSW 2145, Australia; SMcIntyre@cerebralpalsy.org.au (S.M.); catherine.king@sydney.edu.au (C.K.); harunor.rashid@health.nsw.gov.au (H.R.); 3 CSF Global, Dhaka 1212, Bangladesh; arda.jahan89@gmail.com (I.J.); sultana.risad@gmail.com (R.S.); 4 Asian Institute of Disability and Development, University of South Asia, Dhaka 1213, Bangladesh; 5 School of Health, Medical and Applied Sciences, Central Queensland University, Rockhampton, QLD 4701, Australia; 6 Cerebral Palsy Alliance, The University of Sydney, Sydney, NSW 2050, Australia; nadia.badawi@health.nsw.gov.au; 7 Department of Family and Community Medicine, Faculty of Medicine in Rabigh, King Abdulaziz University, Jeddah 22252, Saudi Arabia; ambadahdah@kau.edu.sa; 8 Department of Physiotherapy, School of Rehabilitation Sciences, The University of Jordan, Amman 11942, Jordan; nihadAA@gmail.com; 9 Marie Bashir Institute for Infectious Diseases and Biosecurity, School of Biological Sciences and Sydney Medical School, University of Sydney, Westmead, NSW 2145, Australia; 10 Grace Centre for Newborn Intensive Care, Sydney Children’s Hospital Network, Westmead, NSW 2145, Australia; 11 Discipline of Child and Adolescent Health, Sydney Medical School, The University of Sydney, Sydney, NSW 2145, Australia; 12 Central Queensland Public Health Unit, Central Queensland Hospital and Health Service, Rockhampton, QLD 4700, Australia

**Keywords:** Arabic-speaking countries, malnutrition, children, adolescents, cerebral palsy

## Abstract

Background: We aimed to estimate the burden and underlying risk factors of malnutrition among children and adolescents with cerebral palsy in Arabic-speaking countries. Methods: OVID Medline, OVID Embase, CINAHL via EBSCO, Cochrane Library, and SCOPUS databases were searched up to 3 July 2021. Publications were reviewed to identify relevant papers following pre-defined inclusion/exclusion criteria. Two reviewers independently assessed the studies for inclusion. Data extraction was independently completed by two reviewers. Descriptive and pooled analysis has been reported. Results: From a total of 79 records screened, nine full-text articles were assessed for eligibility, of which seven studies met the inclusion criteria. Study characteristics, anthropometric measurements used, and nutritional outcome reported varied between the studies. The included studies contained data of total 400 participants aged 1–18 years. Overall, (mean: 71.46%, 95% confidence interval: 55.52–85.04) of children with cerebral palsy had at least one form of malnutrition. Severe gross motor function limitation, feeding difficulties, cognitive impairment and inadequate energy intake were the commonly reported underlying risk factors for malnutrition among children with cerebral palsy. Conclusions: The burden of malnutrition is high among children with cerebral palsy in Arabic-speaking countries. More research is needed for better understanding of this public health issue in these countries.

## 1. Introduction

Cerebral palsy (CP) is considered as one of the leading causes of motor disability among children and adolescents [1]. Malnutrition is defined as a person’s energy and/or nutritional consumption being deficient, excessive, or unbalanced. Malnutrition has a broad definition that refers to two types of problem. First, stunting (low height for age), wasting (low weight for height), underweight (low weight for age), and micronutrient deficiencies or insufficiencies are some of the symptoms of undernutrition (a lack of important vitamins and minerals). Second, overweight, obesity, and noncommunicable diseases linked to diet are the other two (such as heart disease, stroke, diabetes, and cancer) [2]. Malnutrition can be seen as a secondary health issue that can impact on the overall health and well-being of children with CP and their families [3]. It occurs when food intake falls short of the requirements for normal body functions, causing growth and development problems [4]. Malnutrition must be diagnosed, prevented, and managed early in children’s lives because growth and development depend on optimum nutritional intake. Malnutrition in children with a chronic condition such as CP is caused by various factors, including the underlying disorder and non-illness-related factors such as increased caloric demands, malabsorption, altered nutrient use, and nutrient provision limits due to fluid status and/or feeding tolerance [5].

There are many ways to evaluate malnutrition and related risk factors among children, including, but not limited to, standard anthropometric measures like weight and its percentile, height and its percentile, body mass index (BMI), waist, head, and arm circumferences. Other measurements that could be used are total body water, fat mass, triceps fold thickness, z-score, and biochemical parameters such as hemoglobin, ferritin, and albumin [4,6].

Despite differences among Arabic-speaking countries (ASCs) (Table 1) in the quality of health care provided, they share many common customs in relation to cultural, social, and food habits. Regardless of these similarities and differences, children with CP are equally vulnerable to malnutrition, yet the burden of malnutrition among children and adolescents with CP in these countries has not been quantified through a systematic review.

Because of the dearth of knowledge regarding the nutritional status of children with CP from ASCs, and to advance the global knowledge base on this crucial issue, we aimed to estimate the burden and underlying risk factors of malnutrition among children and adolescents with CP in the ASCs based on available published literature, to facilitate evidence-based medicine. We realize the need for systematic data collection and reporting of the limited available studies. In this review, therefore, we focused on summarizing the available information regarding the size of the problem and its causes, despite the scarcity of available resources that could be used to conduct large scale studies and nutrition intervention in a similar context.

## 2. Materials and Methods

For this review, we followed the Preferred Reporting Items for Systematic Reviews and Meta-Analysis (PRISMA) guidelines on conducting systematic reviews, including the 27-item checklist [7,8].

### 2.1. Data Sources and Search Strategy

We identified 22 countries whose official language is Arabic [9]. One author (C.K.) searched the following bibliographic databases—OVID Medline (1946–25 June 2021), OVID Embase (1947–1 July 2021), CINAHL via EBSCO (1982–July 2021), Cochrane Library Database of Systematic Reviews (Issue 7 of 12, 2021), Cochrane Central Register of Controlled Trials (Issue 7 of 12, 2021) and SCOPUS (1788–July 2021) to find publications on nutritional status among children and adolescents with CP in ASCs. The final search was completed on 3 July 2021. No language or date limits were applied to ensure maximum retrieval.

The search used controlled vocabulary terms including ‘Cerebral Palsy’, ‘Nutritional status’, ‘Nutritional Sciences’, ‘Malnutrition’, ‘Thinness’, ‘Growth disorders’, ‘Cachexia’, ‘Body Mass Index’, ‘Overweight,’ ‘Obesity’, “Infant Newborn, ‘Infant’, ’Child Preschool’, ‘Child’ and ‘Adolescent’. These were used with corresponding text-word terms. Text-word terms were truncated where necessary to include all relevant term endings. The search terms were combined with the individual country list terms provided in Table 1. The Ovid Medline search strategy used is provided in Appendix A.

### 2.2. Study Selection and Inclusion

Study selection was completed following a pre-set eligibility criteria developed by three reviewers (G.K., S.M., & S.M.M.). The inclusion criteria were as follows: (1) studies reported original observations (from observational and analytical study design); (2) study participants were children and/or adolescents with CP aged up to 18 years in ASCs; and (3) studies reported malnutrition (i.e., underweight, or overweight) as an outcome or in the background characteristics.

The exclusion criteria were as follows: (1) studies reporting a single case, case series, non-observational studies (e.g., systematic reviews, narrative reviews, scoping reviews), conference reports/posters, (2) study participants were only malnourished children or adults with CP, (3) conducted in non-Arabic speaking countries.

Two reviewers, (S.M.M. and G.K.) independently reviewed the identified studies and disagreements were resolved by a third reviewer (I.J.) by consensus. The review protocol has been registered in PROSPERO (registration number: CRD42021244171—https://www.crd.york.ac.uk/prospero/display_record.php?RecordID=244171—accessed on 27 July 2021).

### 2.3. Risk of Bias Assessment

We assessed the selected studies to identify risk of bias using the Newcastle-Ottawa Quality Assessment Scale (NOS) [10]. The assessment was completed by the first author (S.M.M.) with support of an external reviewer (H.B.). Results of individual studies included in this review are shown in Table 2. All seven articles included in this review displayed good quality in all three areas of the assessment (i.e., selection, comparability, and outcome). None of the studies were excluded due to poor quality at this stage as all of them met the standard thresholds for inclusion.

### 2.4. Data Extraction

Data extraction was completed in an Excel templated developed by the first author (S.M.M.) in consultation with another two reviewers (G.K. and I.J.). Two reviewers (R.S. and I.J.) completed data extraction from all seven studies independently. Any differences identified were resolved following discussion with a third reviewer (G.K.). As the most commonly utilized method reported in the studies was anthropometric measurements, the following were extracted as available: (i) study characteristics (citation, implementation country, study settings, study design, study participants, samples size, age and gender, study duration), (ii) outcome measures/measurements used (anthropometric, biochemical, others), (iii) outcome reported (malnutrition proportions and significantly associated risk factors). If any information was unavailable, then it was documented as ‘not reported’.

### 2.5. Data Analysis

Descriptive information (e.g., study characteristics and outcome measures) were presented in table format. The rate of malnutrition was reported as documented in the original study. Factors related to malnutrition reported in individual studies were also summarized, but the effect size could not be estimated due to lack of consistent data. Furthermore, a forest plot and a funnel plot showing the proportion (with 95% confidence interval (CI)) of at least one form of malnutrition as reported in individual studies were constructed. For studies where malnutrition rate was reported for multiple indicators, the highest proportion was included. For meta-analysis, we used MedCalc® Statistical Software version 20.009 (MedCalc Software Ltd., Ostend, Belgium; https://www.medcalc.org; accessed on 20 July 2021). To investigate the heterogeneity we used a random effect model in the analysis. Heterogeneity was considered mild if *I*^2^ < 30%, moderate if *I*^2^ = 30–50%, and notable if *I*^2^ > 50%.

## 3. Results

### 3.1. Study Characteristics and Participants

**Figure 1:**
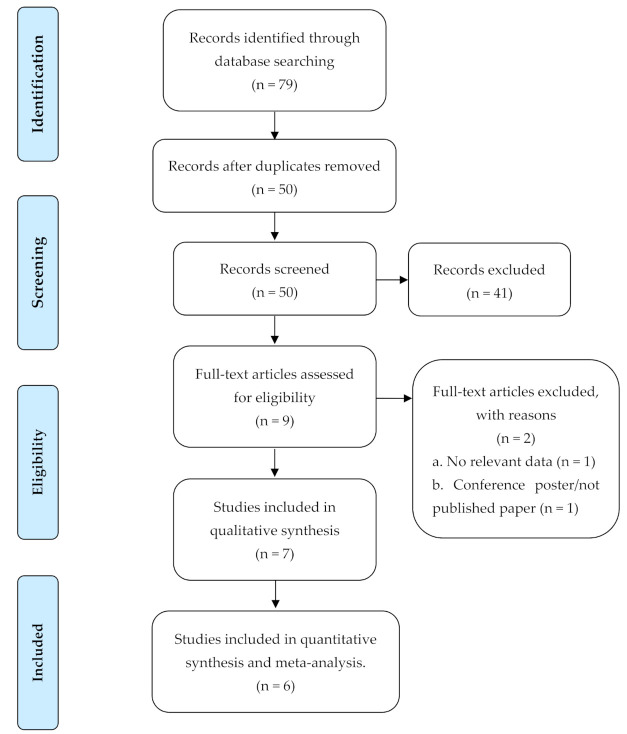
PRISMA flow diagram for a systematic literature review on malnutrition among children and adolescents with CP in ASCs.

A total of 79 titles were identified from the databases following the search strategy described above. After deduplication using EndNoteX9 citation manager and a manual re-check, 50 primary studies were identified of which 41 irrelevant studies were excluded and nine studies were eligible for full-text review. Following consensus among the reviewers, seven articles were selected for inclusion and data extraction. The details have been summarized in Figure 1.

Table 3 summarizes the characteristics of the included studies (*n* = 7). The studies were published between 1984 and 2021 and in English language [4,11,12,13,14,15,16]. Most of the studies included were from Saudi Arabia (*n* = 4) [4,11,12,16], and the remaining were from Egypt (*n* = 1) [13], United Arab Emirates (*n* = 1) [14], and Jordan (*n* = 1) [15]. The study designs of the included studies were cross-sectional (*n* = 5) [4,11,13,14,15], retrospective record review (*n* = 1) [12], and in one study the design was not clearly mentioned [16]. Five studies were hospital/institution-based [4,12,13,15,16], one was school-based [11] and one was a population-based study [14]. Studies differed in terms of duration, sample size, causes of malnutrition, assessment measures used, and nutritional indicators reported.

Overall, two studies were conducted among children with CP only [4,12], whereas, the remaining studies included children with CP as part of a larger cohort of children with disability (*n* = 2) [12,15], special needs (*n* = 1) [11], or compared with control groups (*n* = 2) [13,15]. The total of 400 pooled participants ranged from 12 to 119 children with CP in each study, whose age ranged between 1–18.4 years. Male-female numbers/percentages were available for five of seven studies, which ranged between 47% to 58.3% males, and 41% to 53% females.

### 3.2. Measurements Used for Nutritional Assessment

Among reported nutritional assessment indicators used, all studies used at least one standard anthropometric measurement tool. Most commonly reported indicators were percentiles/z-scores for weight-for-age (*n* = 3) [4,12,13], height for age (*n* = 2) [4,13], and BMI/BMI-for-age (*n* = 3) [4,11,14]. Additionally, body composition and biochemical tests were reported in one study [13] as an indicator for nutritional status. The nutritional indicators reported in the included studies have been summarized in Table 4.

### 3.3. Malnutrition Rate among Children with CP

**Figure 2:**
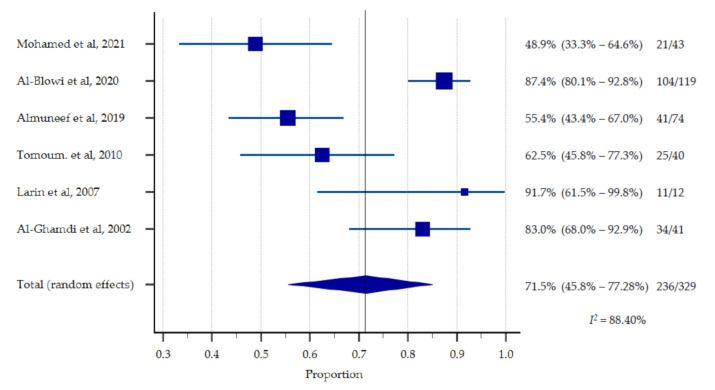
Presence of at least one form of malnutrition (%).

**Figure 3:**
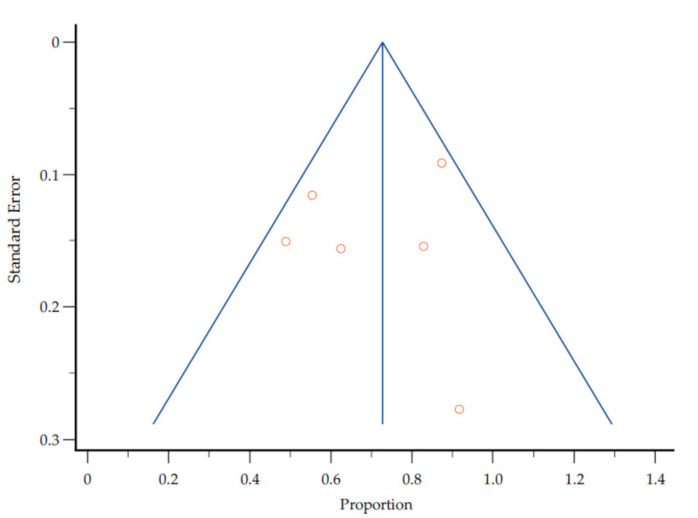
Assessment of publication bias by a funnel plot of the proportion of malnutrition estimates against corresponding standard error.

Out of a total N = 952 participants in the included studies, *n* = 400 were children with CP and were eligible for estimation of the pooled prevalence of malnutrition. However, the proportion of at least one form of malnutrition among children with CP was reported in *n* = 6 studies [4,11,12,13,14,16] whereas the mean (SD) nutritional indicator was reported in *n* = 2 studies [14,15]. The pooled estimates suggest that 48.84–91.67% children with CP in the included studies had at least one form of malnutrition (pooled prevalence of 71.46%, 95% CI: 55.52–85.04, *p* < 0.0001). Moderate to severe underweight was most frequently reported (*n* = 4) and ranged between 7%–84.9% among the participating children with CP [4,11,12,13]. Being overweight was reported in *n* = 3 studies and ranged between 2.5–25% [11,12,14] (Table 5, Figure 2 and Figure 3).

### 3.4. Underlying Risk Factors of Malnutrition

Five out of seven included studies reported the factors related to malnutrition among children with CP in ASCs [4,11,12,13,14]. Overall, malnutrition was found higher among children with moderate-severe gross motor function limitation (e.g., GMFCS level III–V) [13,15], oro-motor dysfunction/feeding difficulties [12,13], with traumatic dental injury, caries and medical complications [11,12]. Furthermore, older age of the child, presence of cognitive impairment and inadequate energy intake were reported as contributing factors to malnutrition among children with CP in one study [4].

### 3.5. Study Quality and Heterogeneity

The symmetrical funnel plot in Figure 3 revealed that there was no substantial publication bias in the meta-analysis (Figure 2) for the proportion of malnutrition estimates against corresponding standard error. However, there is a high clinical heterogeneity (*I*^2^ = 88.40%) as the included studies did not use uniform measurements of malnutrition.

## 4. Discussion

To the best of our knowledge, this is the first systematic review reporting the burden of malnutrition and its underlying risk factors among children and adolescents with CP in ASCs. In our review we observed that the burden of malnutrition among children with CP in ASCs is obviously understudied. Although we included all 22 countries during our detailed search, the results yielded studies from only four countries. Furthermore, most studies were conducted in institution-based settings (e.g., hospitals, health care facilities, schools) limiting the opportunities to generalize the findings. This indicates an urgent need for more medical research on this crucial issue, especially in the setting of low-to-middle income countries (LMIC). Although most of the ASCs are classified as low or middle income, with the exception of the Gulf countries [17], among the included studies in our review only two (out of seven) were from LMIC settings (e.g., Egypt, Jordan) [13,15]. More research is needed to investigate the factors that contribute to this evidence gap.

The indicators used/type of malnutrition reported varied substantially between the studies and sufficient data were not available to estimate the pooled prevalence of different types of malnutrition (e.g., underweight, stunting, overweight, wasting, etc.). Hence, we reported the pooled proportion of at least one form of malnutrition among participating children with CP in the Arabic-speaking countries. Nevertheless, the overall malnutrition rate was high among children with CP in ASCs, especially when compared to children without CP.

Being underweight was the most commonly reported form of malnutrition, although the proportions varied substantially between countries. However, when compared to other institution-based studies, the proportion of undernutrition was higher in Arabic-speaking LMICs (e.g., Egypt) than non-Arabic-speaking LMICs (e.g., Vietnam and Argentina) [18,19,20]. We also observed a wide range of overweight/obesity among the participating children in the included studies.

Malnutrition in children with disabilities, including CP, could be due to several interlinked underlying risk factors which varies from one population to another [20]. Only a few of the included studies reported the underlying factors, of which gross motor function and feeding difficulties were predominant [12,13,15]. Although we could not measure the effect size of these underlying factors on malnutrition rate, due to the heterogeneity in the reported data (*I*^2^ = 84.40%), it is known that gross motor function significantly affects nutritional status and is closely related to the presence and severity of feeding difficulties among children with CP [21,22]. Children with higher gross motor impairment therefore require careful evaluation and nutritional intervention to improve their nutritional as well as functional outcome [20]. One study also reported inadequate energy intake as an influencing factor of malnutrition among the participating children [4]. This relationship is straightforward, but the reason for lack of energy consumption could be due to clinical factors or lack of access to resources. All these findings indicate that there is an urgent need to generate robust data to identify the modifiable causes and a potential practical intervention relating to these crucial issues among children with CP in ASCs. Malnutrition among children with CP is a major concern. It is often associated with a number of other comorbidities. Iron deficiency anaemia (IDA), renal impairment, auditory and visual deficiency, low bone mineral density, poor growth, and infections have been reported in previous studies [4,12,14,15,16,23].

This review has some limitations which are evident in the small number of studies (seven for 22 countries), so not all countries are represented. Thence, we did not exclude any studies based on the CP definition. However, for outcome measures such as undernutrition or overnutrition, we used standard criteria. For instance, underweight was defined as a child’s weight-for-age being ≤2SD or 15th percentile.

Although we conducted a comprehensive search, the number of studies identified was very small, indicating that there is a large gap in the evidence in ASCs in this regard. This is one of the main reasons why this review assesses and maps the existing evidence to generate comprehensive data on the nutritional status of children with CP in ASCs.

In addition, there was high clinical heterogeneity, non-uniform anthropometric measurements, and the age group ranged up to 18.4 years in one study [14], although one of the inclusion criteria was up to 18 years old. The included studies were mostly conducted in institution-based settings, hence the pooled estimates are not generalizable. We could not estimate the effect size of different underlying factors on nutritional status of children with CP in ASCs, although this was one of our study objectives. Furthermore, malnutrition can take several forms, including underweight and/or overweight. However, because anthropometric measurements are the most commonly used method, and the majority of studies reporting nutritional status of children used those terminologies, we only focused on nutritional status reported based on anthropometric measurements. Nevertheless, the strength of this review is that it is a uniquely novel systematic review and meta-analysis on an under-researched theme. It addresses a very important public health issue involving children with disability-like CP. All of the studies included are of good quality with a symmetrical funnel plot.

## 5. Conclusions

Malnutrition in children and adolescents with disabilities and/or CP is an existing problem in ASCs but there is a dearth of medical research. Focused research is needed to fill the large evidence gap and identify need-based effective nutrition intervention for children with CP in these countries.

## Figures and Tables

**Table 1:** The Arabic-speaking countries in alphabetical order.

Country	Official Name	Total Populations *	Population Aged ≤ 19 years * (%)
Algeria	People’s Democratic Republic of Algeria	43,851,043	16,409,237 (37)
Bahrain	Kingdom of Bahrain	1,701,583	399,990 (24)
Comoros	Union of the Comoros	869,595	428,906 (49)
Djibouti	Republic of Djibouti	988,002	376,430 (38)
Egypt	Arab Republic of Egypt	102,334,403	43,413,971 (42)
Emirates	United Arab Emirates	9,890,400	1,854,704 (19)
Iraq	Republic of Iraq	40,222,503	19,320,987 (48)
Jordan	Hashemite Kingdom of Jordan	10,203,140	4,392,416 (43)
Kuwait	State of Kuwait	4,270,563	1,141,552 (27)
Lebanon	Lebanese Republic	6,825,442	2,287,154 (34)
Libya	State of Libya	6,871,287	2,471,165 (36)
Mauritania	Islamic Republic of Mauritania	4,649,660	2,315,383 (50)
Morocco	Kingdom of Morocco	36,910,558	12,849,811 (35)
Oman	Sultanate of Oman	5,106,662	1,362,877 (27)
Palestine	State of Palestine	5,101,416	2,474,021 (48)
Qatar	State of Qatar	2,881,060	498,936 (17)
Saudi Arabia	Kingdom of Saudi Arabia	34,813,867	10,816,497 (31)
Somalia	Federal Republic of Somalia	15,893,219	9,152,954 (58)
Sudan	Republic of Sudan	43,849,269	22,252,463 (51)
Syria	Syrian Arab Republic	17,500,657	6,961,028 (40)
Tunisia	Republic of Tunisia	11,818,618	3,657,697 (31)
Yemen	Republic of Yemen	29,825,968	14,783,682 (50)

* Estimates, 2020, https://population.un.org/wpp/Download/Standard/Poplation/ (accessed on 8 July 2021).

**Table 2:** Newcastle-Ottawa Scale (NOS) scores for included studies.

Author	Selection Max 4 Stars	Comparability Max 2 Stars	Outcome Max 3 Stars	Score * Max 9 Stars
Mohamed et al., 2021 [11]	★★★★	★★	★★★	9
Al-Blowi et al., 2020 [12]	★★★	★	★★★	7
Almuneef et al., 2019 [4]	★★★★	★★	★★★	9
Tomoum. et al., 2010 [13]	★★★★	★	★★★	8
Larin et al., 2007 [14]	★★★	★	★★	6
Ibrahim et al., 2007 [15]	★★★	★★	★★★	8
Al-Ghamdi et al., 2002 [16]	★★★	★	★★	6

* Thresholds for converting the Newcastle-Ottawa scales to AHRQ standards (good, fair, and poor): Good quality: 3 or 4 stars in selection domain AND 1 or 2 stars in comparability domain AND 2 or 3 stars in outcome/exposure domain. Fair quality: 2 stars in selection domain AND 1 or 2 stars in comparability domain AND 2 or 3 stars in outcome/exposure domain. Poor quality: 0 or 1 star in selection domain or 0 stars in comparability domain or 0 or 1 stars in outcome/exposure domain.

**Table 3:** Summary of characteristics of included studies.

ID	Authors	Country	Study Period	Study Design	Study Settings	Study Population	Sample Size	Age of Participants	Female: Male (%)
1.	Mohamed et al., 2021 [11]	Saudi Arabia	Sep 2018–Mar 2019	Cross-sectional	School based	Children with special health care needs including CP	N = 400, CP: *n* = 43	Range: 6–16 years	CP information not reported
2.	Al-Blowi et al., 2020 [12]	Saudi Arabia	2012–2016	Retrospective record review	Hospital/institution-based	Children with CP	N = 119	Mean (standard deviation [SD]): 5.9 (3.8) years	F: 53.0; M: 47
3.	Almuneef et al., 2019 [4]	Saudi Arabia	Jan–Aug 2015	Cross-sectional	Hospital/institution-based	Children with CP	N = 74	Range: 1–12 years	F: 41.0; M: 59
4.	Tomoum. et al., 2010 [13]	Egypt	Apr–Oct 2007	Cross-sectional	Hospital/institution-based	Children with CP and controls	N = 80, CP: *n* = 40	Range: 2–8 years	F: 47.5; M: 52.5
5.	Larin et al., 2007 [14]	United Arab Emirates	Not reported	Cross sectional	Population based	Children with physical disability including CP	N = 17, CP: *n* = 12	Range: 4.2–18.4 years;mean (SD): 10.4 (4.6) years	F: 41.7; M: 58.3
6.	Ibrahim et al., 2007 [15]	Jordan	Mar 2005–Mar 2006	Cross sectional	Hospital/institution-based	Children with spastic CP and control group without CP	N = 151, CP: *n* = 71	Range: 3–7 years	Not reported
7.	Al-Ghamdi et al., 2002 [16]	Saudi Arabia	1998	Not reported	Hospital/institution-based	Children with disability including CP	N = 111, CP: *n* = 41	Range: 1.1- just over 13 years; Mean (SD): 6.0 (2.7) years	F: 48.2; M: 51.8

**Table 4:** Anthropometric measurements used.

ID	Authors	Anthropometric Measurements Used	Nutritional Indicator Reported
1.	Mohamed et al., 2021 [11]	(i) weight, (ii) height	BMI
2.	Al-Blowi et al., 2020 [12]	(i) weight, (ii) height, (iii) head circumference	weight-for age (underweight)
3.	Almuneef et al., 2019 [4]	(i) weight, (ii) height, (iii) arm circumference, (iv) arm muscle circumference, and (v) triceps skinfold thickness	weight-for age z-score, weight-for-height z-score, height-for-age z-score, BMI-for-age z-score, arm circumference, arm muscle circumference, triceps skinfold thickness
4.	Tomoum. et al., 2010 [13]	(i) body weight, (ii) head circumference, (iii) mid-upper arm circumference, (iv) waist and hip circumferences	(i) weight percentile, (ii) height percentile, (iii) BMI percentile, (iv) hip circumference, (v) waist-to-hip circumference ratio
5.	Larin et al., 2007 [14]	(i) weight, (ii) height	BMI, BMI-for-age percentile
6.	Ibrahim et al., 2007 [15]	(i) stature, (ii) weight, (iii) head circumference, (iv) mid-upper arm circumference	Mean value for each measurement according to motor type of CP
7.	Al-Ghamdi et al., 2002 [16]	(i) weight	weight for height z-score (wasting)

**Table 5:** Key findings of the included studies.

ID	Authors	Sample Size	Proportion of Malnutrition among Children with CP	Factors Related to Nutritional Status
1.	Mohamed et al., 2021 [11]	N = 400, CP: *n* = 43	Underweight: 7%. Overweight: 7%. Obese: 34.9%.	Positive association was found between dental caries and obesity among participating children.
2.	Al-Blowi et al., 2020 [12]	N = 119	Underweight: 84.9%. Overweight: 2.5%.	Underlying factors of undernutrition: feeding difficulty, and medical complications were found associated to malnutrition.
3.	Almuneef et al., 2019 [4]	N = 74	Malnourished: 55.4%. Thinness: 50.0%. Underweight: 28.4%. Stunting: 33.8%. Wasting: 25.0%.	Underlying factors of undernutrition: Age, cognitive impairment, anemia, and inadequate energy intake.
4.	Tomoum. et al., 2010 [13]	N = 80, CP: *n* = 40	Weight percentile < 10th: 14.3% among males, 15.8% among females. Weight percentile < 50th: 38.1% among males, 47.4% among females. Height percentile < 10th: 5.3% among females, 4.8% among males. Height percentile < 50th: 47.7% among males, 78.9% among females.	Overall, children with CP had significantly lower weight, height, head circumference, waist circumference and TST than children without CP. Underlying factors of undernutrition: Gross Motor Function Classification System (GMFCS) level III-V and oro-motor dysfunction.
5.	Larin et al., 2007 [14]	N = 17, CP: *n* = 12	Thinness (i.e., BMI-for-age < 15th percentile): 75%. Overweight (i.e., BMI-for-age z-score 85th- < 97th percentile): 25%. Mean (SD) BMI-for-age z-score: 15.6 (3.5) kg/m^2^.	Not reported.
6.	Ibrahim et al., 2007 [15]	N = 151, CP: *n* = 71	Mean values; Stature (cm): diplegic—males 99.6, females 96.1; quadriplegic—males 92.0, females 92.0; hemiplegic—males 102.4, females 94.0. Weight (kg): diplegic—males 15.1, females 15; quadriplegic—males 12.6, females 11.2; hemiplegic—males 17.1, females 16.5. Head circumference (cm): diplegic—males 49.6, females 49.4; quadriplegic—males 45.7, females 46.3; hemiplegic—males 52.1, females 41.5. Arm circumference (cm): diplegic—males 17.2, females 17.0; quadriplegic—males 15.7, females 14.7; hemiplegic—males 18.0, females 16.3.	Overall, stature, weight, head circumference and MUAC of children with CP were significantly lower compared to the control groups. Weight was significantly associated with gross motor function.
7.	Al-Ghamdi et al., 2002 [16]	N = 111, CP: *n* = 41	Severely malnourished: 83%.	Not reported.

## Database

Ovid MEDLINE(R) ALL <1946 to 25 June 2021>.

## Appendix A

Ovid MEDLINE search strategy:

**Table A1:** Search Strategy.

1	exp Cerebral Palsy/
2	(cerebral adj pals$).tw.
3	CP.tw.
4	1 or 2 or 3
5	exp Nutritional Status/
6	exp Nutritional Sciences/
7	nutriti$.tw.
8	exp Malnutrition/
9	(malnutrition$ or malnourish$).tw.
10	undernourish$.tw.
11	undernutriti$.tw.
12	exp Thinness/
13	(thin$ or lean$ or underweight$).tw.
14	exp Growth Disorders/
15	(grow$ adj5 disorder$).tw.
16	stunt$.tw.
17	exp Cachexia/
18	(cachexia$ or wasting or wasted).tw.
19	exp Body Mass Index/
20	(“body mass index$” or BMI).tw.
21	“anthropometric measur$”.tw.
22	exp Overweight/
23	exp Obesity/
24	(overweight or obese or obesit$).tw.
25	5 or 6 or 7 or 8 or 9 or 10 or 11 or 12 or 13 or 14 or 15 or 16 or 17 or 18 or 19 or 20 or 21 or 22 or 23 or 24
26	4 and 25
27	exp Jordan/
28	jordan$.tw.
29	exp United Arab Emirates/
30	emirate$.tw.
31	uae.tw.
32	exp Bahrain/
33	bahrain$.tw.
34	exp Tunisia/
35	tunisia$.tw.
36	exp Algeria/
37	algeria$.tw.
38	exp Djibouti/
39	djibouti$.tw.
40	exp Saudi Arabia/
41	(saudi adj1 arabia$).tw.
42	exp Sudan/
43	sudan$.tw.
44	exp Syria/
45	syria$.tw.
46	exp Somalia/
47	somalia$.tw.
48	exp Iraq/
49	iraq$.tw.
50	exp Oman/
51	oman$.tw.
52	palestin$.tw.
53	exp Qatar/
54	qatar$.tw.
55	exp Comoros/
56	comoros$.tw.
57	exp Kuwait/
58	kuwait$.tw.
59	exp Lebanon/
60	leban$.tw.
61	exp Libya/
62	libya$.tw.
63	exp Egypt/
64	egypt$.tw.
65	exp Morocco/
66	morocco$.tw.
67	exp Mauritania/
68	mauritania$.tw.
69	exp Yemen/
70	yemen$.tw.
71	exp Arabs/
72	(arab$ adj4 (speak$ or countr$ or world)).tw.
73	27 or 28 or 29 or 30 or 31 or 32 or 33 or 34 or 35 or 36 or 37 or 38 or 39 or 40 or 41 or 42 or 43 or 44 or 45 or 46 or 47 or 48 or 49 or 50 or 51 or 52 or 53 or 54 or 55 or 56 or 57 or 58 or 59 or 60 or 61 or 62 or 63 or 64 or 65 or 66 or 67 or 68 or 69 or 70 or 71 or 72
74	26 and 73
75	limit 74 to "all child (0 to 18 years)"
76	exp Infant, Newborn/
77	exp Infant/
78	exp Child, Preschool/
79	exp Child/
80	exp Adolescent/
81	(baby or babies or infant$ or toddler$ or child$ or adolescen$ or pediatric$ or pediatric$).tw.
82	76 or 77 or 78 or 79 or 80 or 81
83	74 and 82
84	75 or 83
